# Postnatal Growth Disadvantage of the Small for Gestational Age Preterm Twins

**DOI:** 10.3390/nu10040476

**Published:** 2018-04-12

**Authors:** Iris Morag, Orly Stern Levkovitz, Maya Siman-Tov, Mor Frisch, Orit Pinhas-Hamiel, Tzipi Strauss

**Affiliations:** 1 Sackler School of Medicine, Tel Aviv University, Tel Aviv 6997801, Israel; orly.stern77@gmail.com (O.S.L.); morf@mail.tau.ac.il (M.F.); Orit.Hamiel@sheba.health.gov.il (O.P.-H.); t.tzipi@gmail.com (T.S.); 2 Neonatology Department, Edmond and Lily Safra Children’s Hospital, Sheba Medical Center, Tel Ha’Shomer, Ramat Gan 52621, Israel; 3 Gertner Institute for Epidemiology and Health Policy, Sheba Medical Center, Ramat Gan 52621, Israel; maylev90@gmail.com; 4 Pediatric Endocrine and Diabetes Unit, Edmond and Lily Safra Children’s Hospital, Sheba Medical Center, Ramat Gan 52621, Israel

**Keywords:** preterm infant, twin, small for gestational age, growth

## Abstract

In this study, we examined early growth characteristics among small-for-gestational-age (SGA) preterm twins compared to their appropriate-for-gestational-age (AGA) counterparts. A retrospective study evaluated all consecutive twins born between 2008 and 2015 at a tertiary referral center whose gestational age ranged from 30.0 to 34.86 weeks. Included were twins in which one twin was AGA and the other SGA at birth. Changes of ≥2, 1–1.99, and 0–0.99 in z-score between births and 36 weeks post menstrual age (PMA) were respectively defined as severe, moderate, and mild postnatal growth failure (PNGF) in weight or head circumference (HC). Early neonatal morbidities were documented. Multiple logistic regression analysis was applied to determine conditions associated with PNGF and its severity. Out of 666 sets of twins, 83 met the inclusion criteria. Weight PNGF was similar and mild among the SGA and the AGA groups (0.9 ± 0.46 vs. 0.96 ± 0.44 z-score, respectively, *p* = 0.24). At 36 weeks PMA, a significantly larger proportion of SGAs were below −2 z-scores in weight (84.3%) compared to birth (31.3%) or to the AGAs (8.4%). In both groups, weight PNGF correlated with the time needed to regain birth weight. HC PNGF was mild among both groups, yet significantly more prominent among the AGAs (0.39 ± 0.72 z-score) vs. SGAs (0.75 ± 0.65 z-score, *p* = 0.001). We suggest that among preterm SGA infants, the absolute z-score should be used to assess the severity of weight PNGF. Individual nutritional strategies to decrease time to regain birth weight may mitigate severe malnutrition among SGAs.

## 1. Introduction

The condition of being born small for gestational age (SGA) is commonly defined as birth weight below the 10th percentile and occurs in approximately 13% to 20% of all preterm infants [1]. While some of these infants represent constitutionally small fetuses, others represent a subgroup of growth restricted fetuses that did not meet their genetic potential due to various causes, among them multiple pregnancies, placental insufficiency, intra-uterine infections, congenital anomalies, and genetic disorders [1,2]. 

Postnatal growth failure (PNGF) is classically referred to as a condition in which extrauterine growth is less than expected based on intrauterine growth measurements. Infants born SGA as well as those born prematurely are at increased risk for PNGF, mostly due to more severe postnatal weight loss, early neonatal morbidities, and longer time needed to regain their birth weight (BW) and to establish full enteral feeding compared to appropriate-for-gestational-age (AGA) term infants [3,4,5,6,7].

A unique group at high risk for weight PNGF and impaired long-term outcomes encompasses those born premature and SGA [8,9,10]. Early catch-up growth among term SGA infants was associated with beneficial developmental outcomes compared to those who failed to catch up [11,12,13,14,15].

In the present study, we sought to characterize early growth trajectories of preterm SGA infants by comparing them to their AGA born twins. Comparison between twins decreases the effect of other confounders such as genetic, constitutional, or maternal conditions and allows for better understanding of the pure effect of being born SGA on early postnatal growth. It may also help in better understanding the optimal time for intervention.

## 2. Methods

### 2.1. Study Design

The present study included surviving preterm twins born at Chaim Sheba Medical Center in Israel during the years 2008–2015. Gestational age (GA) in completed weeks was determined by the best obstetric estimate of GA based on last menstrual period and on prenatal ultrasound. Postmenstrual age (PMA) was calculated by GA plus chronological age in weeks. Weight and head circumference (HC) at delivery and at 36 weeks GA were documented and converted to z-scores and centiles according to sex-specific growth curves [16]. The Fenton growth charts for preterm infants allow for comparisons to be made between an infant’s growth with its intrauterine growth rate [16]. Small for gestational age (SGA) was considered as birth weight below the 10th centile for GA age. AGA was considered as birth weight between 10th and 90th centile. Head circumference measurements at birth and weekly thereafter until 36 weeks PMA are routinely performed using measuring tape and have been shown to be reliable [17]. The changes in weight and HC z-scores from birth to 36 weeks PMA (∆ z-score) were calculated for each infant by subtracting the z-score at birth from the z-score at 36 weeks PMA. Based on the findings of Shah et al., mild, moderate, and severe PNGF were defined as decreases in z-scores of 0–0.99, 1–1.99, and ≥2, respectively [18].

Included in the study were only those pairs of twins in which one was SGA at birth and the other was AGA according to the above criteria. Excluded were infants who died prior to 36 weeks PMA and those with suspected or confirmed genetic syndromes, as they represent a different group in terms of early growth. In an attempt to keep the cohort as homogenous as possible, those infants born prior to 30 weeks were also excluded due the small number found in the cohort (two set of twins only). Infants’ medical data were collected retrospectively from the computerized medical charts (MetaVision-MDSoft) using a specific data collection form and included the following: GA, maternal hypertension, delivery mode, gender, maternal age, in vitro fertilization, previous pregnancies and deliveries, use of prenatal steroids, Apgar score at 1 and 5 min, body temperature upon admission, need for mechanical ventilation, surfactant treatment, duration of oxygen treatment, oxygen at 28 days, sepsis (diagnosed by positive blood culture), intraventricular hemorrhage (IVH) grade III-IV or periventricular leukomalacia (PVL, diagnosed by the pediatric radiologist through head ultrasound), retinopathy of prematurity requiring treatment (diagnosed by the pediatric ophthalmologist), proven necrotizing enterocolitis (NEC) according to revised Bell’s criteria [19], any surgery during hospitalization, time (days) to regain birth weight, duration of intravenous line (IV), and duration of total parenteral nutrition (TPN). Due to the relatively small number of infants included in each type of morbidity, we defined “severe morbidities” as any of the following: oxygen requirement at 36 weeks GA, NEC stage ≥ 2, IVH grade ≥ 3, PVL, surgical procedure, or sepsis.

The infants included in the study were all cared for in a single neonatal intensive care unit (NICU) from birth until their discharge home. The hospital admits around 10,000 deliveries a year. All infants born <35 weeks and <1.9 kg are admitted to the NICU or the step-down unit, which share the same facility and the same medical team. During the study period, early enteral nutrition was the standard of care for AGA infants, while SGAs were kept nil per os for the first 72 h as a strategy to prevent NEC. These infants were treated with total parenteral nutrition for the first 72 h, followed by enteral nutrition that was gradually increased (20 mL/kg/day) up to a total intake of 150–160 mL/kg/day. Feeding with mother’s own maternal breastmilk was encouraged. Otherwise, preterm infant formula was given because a donor milk bank was not available in the country. Discharge criteria required all of the following: reaching 36 weeks PMA, weighing 1900 g or more, temperature stability, consistent weight gain, full oral feeding, and being free of apneic episodes for >72 h.

This study was approved by the Institutional Review Board 2403-15-SMC. The Hospital Ethics Committee waived the need for informed consent as this was an observational audit of normal practice.

### 2.2. Statistics

Data were analyzed using SPSS statistical software (IBM Corp. Released 2016, IBM SPSS Statistics for Windows, Version 24.0, IBM Corp., Armonk, NY, USA). AGA and SGA infants were compared by paired sample *t*-tests for continuous variables and McNemar’s test for categorical ones. Independent sample *t*-test and Chi-square were used for sub-analyses. Multiple logistic regression analysis was conducted to explore the risk factors for PNGF adjusted for maternal and infant characteristics. Only factors that were significant in the univariate analysis were entered into the regression analysis. A value of *p* < 0.05 was considered statistically significant.

## 3. Results

**Figure 1:**
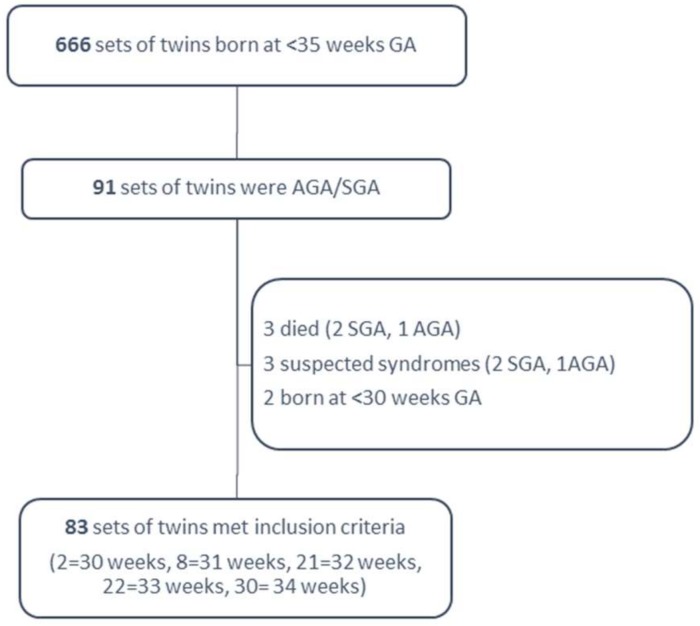
Study population. wks: weeks; GA: gestational age; AGA: appropriate for gestational age; SGA: small for gestational age.

Identified were 666 sets of twins born during the study period at <35 weeks of GA, of whom 91 (13.5%) were AGA/SGA. Eight sets of twins were excluded: three due to neonatal death (2 SGA, 1 AGA) prior to discharge, three for suspected syndromes (2 SGA, 1 AGA), and two born at <30 weeks of gestation. Eighty-three set of twins were entered into the statistical analysis, as showed in Figure 1.

Table 1 shows maternal and pregnancy characteristics, and Table 2 provides infants’ clinical characteristics. The SGA twins were significantly more likely to experience early neonatal morbidities, including severe morbidities. The AGAs required longer time to regain their birth weight and were more likely to be treated with oxygen.

### 3.1. Postnatal Weight Characteristics

**Figure 2:**
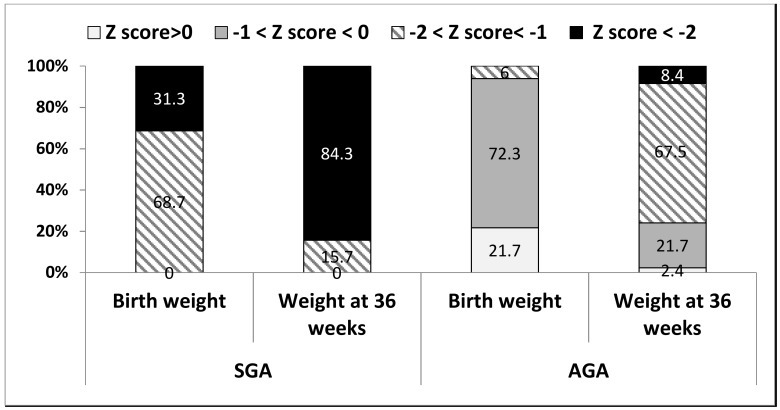
Weigh z-score (absolute) upon admission and at 36 weeks PMA among SGA and AGA (*n* = 83).

Table 3 compares the SGAs to the AGAs in terms of weight parameters. All parameters differed significantly at both time points, except for a similar mild weight PNGF (∆ z-scores of 0.9 ± 0.46 and 0.96 ± 0.44, respectively, *p* = 0.24). Figure 2 demonstrates the distribution of absolute weight z-scores among the groups at birth and at 36 weeks PMA: the proportion of those with <−2 z-scores increased from 31.3% at birth to 84.3% among the SGAs and from 0% to 8.4% among the AGAs.

Multinomial logistic regression was conducted to determine the independent impact of perinatal variables and neonatal morbidities on moderate or severe weight PNGF among the SGA and AGA infants (Table 4). Among SGA infants, an unadjusted logistic regression demonstrated that several early morbidities, such as sepsis, need for oxygen support, and duration of fluid infusion, were associated with higher risk for moderate or severe weight PNGF. Yet after adjusting for GA, BW, and gender, the only factors predicting moderate or severe weight PNGF were GA and days to regain BW, suggesting that lower GA and longer time to regain BW led to a higher risk of moderate or severe weight PNGF among SGA infants. Among the AGAs, an unadjusted logistic regression demonstrated that duration of fluid infusion and days to regain BW were associated with higher risk for moderate or severe weight PNGF. Yet after adjusting for GA, BW, and gender, the only factor predicting moderate or severe weight PNGF was days to regain BW, suggesting that longer time to regain BW led to a higher risk of moderate or severe weight PNGF among AGA infants.

### 3.2. Postnatal Head Circumference Characteristics

**Figure 3:**
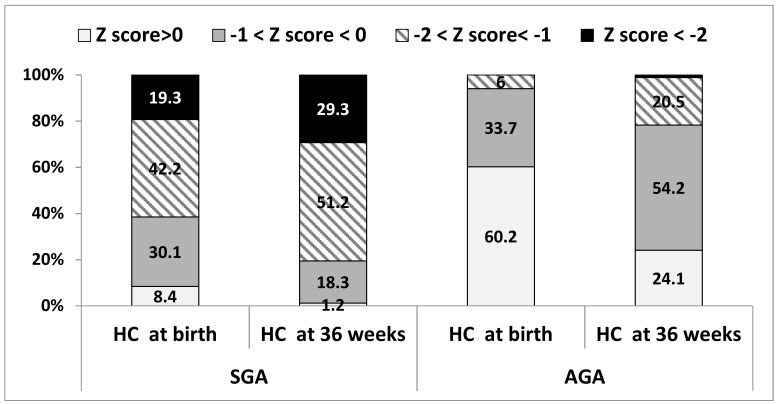
Head circumference Z score change between admission and 36 weeks among AGAs vs. SGAs for total sample (*n* = 83).

HC measures differed significantly between the groups at both time points (Table 3 and Figure 3), including mean HC PNGF (∆ z-score of 0.39 ± 0.72 vs. 0.75 ± 0.66 among SGAs vs. AGAs, respectively, *p* = 0.001). Moderate or severe HC PNGF occurred in 11 (13.3%) of the SGAs compared to 20 (24.4%) of the AGAs. An increase in postnatal HC z-scores occurred in 26.6% of the SGAs and 8.5% of the AGAs. Figure 3 shows the distribution of HC z-scores among the groups at both time points. At 36 weeks PMA, 29% of the SGAs and 1.2% of the AGAs had z-scores below −2, compared to 19.3% and 0% at birth, respectively.

A positive significant correlation between weight and HC PNGF (*r* = 0.319, *p* = 0.003) was found among the AGAs but not among the SGAs. The following were found to be associated with a > 1 z-score decrease in HC among SGAs: younger GA (OR 0.51, 95% CI 0.28–0.93, *p* = 0.028) and lower BW (OR 0.99, 95% CI 0.97–0.99, *p* = 0.020), while among the AGAs only time to regain BW was found (OR 1.18, 95% CI 1.05–1.33, *p* = 0.006).

## 4. Discussion

To the best of our knowledge, this is the first study that used twin data to study the early postnatal growth trajectories of SGA preterm infants. Our results reveal that while the weight of 31.3% of the SGAs at birth was below −2 z-scores, by the time they reached 36 weeks PMA, the weight of 84% of them was below −2 z-scores, compared to <10% of the AGAs. PNGF, however, was mild (∆ z-score < 1) and similar for both twins, and severe PNGF was rare (<4%). These data suggest that the majority of SGA preterm infants continue to regress and suffer extreme malnutrition (as indicated by absolute z-scores) by the time they reach 36 weeks PMA, despite mild PNGF (as indicated by ∆ z scores). If only ∆ z-scores are considered, this extreme may be overlooked. Future studies are required in order to determine which of the measures (absolute z-score vs. ∆ z-scores) has a better predictive value for long-term outcomes among SGA-born preterm infants. Time to regain birth weight was the only modifiable factor associated with weight PNGF, suggesting the potential for early intervention. 

Weight PNGF has been estimated to occur in 30–88% of very preterm infants, depending on the definition used [18,20,21]. Assessing weight PNGF is important, as it has been shown to be a risk factor for adverse long-term neurodevelopmental outcomes [8,22]. Moreover, understanding growth patterns and risks for weight PNGF may help in planning early interventions that have the potential to improve outcomes. Weight PNGF has commonly been considered as growth values <10th percentile of expected intrauterine growth [9,23,24,25] or as a decrease in z-scores between birth and a defined postmenstrual age [18,20,26,27,28,29,30]. Use of the first definition fails to estimate growth failure among those born SGA, as by definition their BW is already <10th centile. In a study that included 74 infants born at ≤28 weeks gestation, Shah et al. found that the best prediction for adverse long-term neurodevelopmental outcome was observed with the measure based on a difference in z-score of >2 between birth and 36 weeks PMA. According to that study, a difference of >2 z-scores during that period occurred in 23 (31%) infants [18]. In the present study, which included more mature preterm infants, severe weight PNGF of >2 z-scores was, as expected, rare among both the SGA and the AGA groups. While both groups experienced a similar mild weight PNGF (∆ z-score < 1), the proportion of SGAs scoring < 2 z-score on their absolute weight more than doubled from birth to 36 weeks PMA. Taken together, these findings suggest the need for further research in assessing the effect of absolute z-score in addition to ∆ z-score when assessing postnatal growth among SGA-born moderate and late preterm infants. Future research is needed to correlate these finding with their long-term outcomes.

One may argue that assessing PNGF at 36 weeks PMA for moderate and late preterm infants may be too early. Although the optimal time point for postnatal growth assessment has not yet been determined, several studies do suggest that early growth trajectories may be of great importance. Studies have shown that in the first two years of life, substantial variability can be observed in infants’ rate of weight gain according to catch-up or catch-down growth periods. In a study by Çamurdan et al., term-born infants already achieved their catch-up growth by the second month of life [31]. Varella et al. assessed 5640 SGA infants and found that patterns of weight gain before four months of age were associated with differences in intelligence quotient IQ scores at four years: IQ scores were significantly lower among those who experienced early catch-down compared to the other groups. The authors concluded that their findings indicate that patterns of weight gain before four months of age may already impact IQ scores at four years of age [15]. In a population-based cohort study (Epiphage) that included all live infants born between 22 and 32 weeks gestation, the authors found that growth velocity within the first 15 days of life was highly associated with growth profile at six months of age. In that study, weight PNGF, defined as loss of ≥1 SD from birth to six months, was significantly associated with cerebral palsy among AGAs and with cognitive deficiency and school difficulties among SGAs, though this was not statistically significant. The optimal time to assess weight PNGF and its correlation with long-term outcomes has yet to be established. Nevertheless, the above studies do indicate that early growth trajectories have an effect on long-term outcomes and should not be overlooked. Early nutritional support, neonatal weight gain, and prevention of early catabolic state have been shown to have a positive impact on the long-term outcomes of preterm infants [8,29,32]. Our findings demonstrate that the time to regain birth weight correlates with severity of weight PNGF at 36 weeks. This modifiable factor may serve as a marker for the need for a different and unique form of early nutritional support for SGA born infants. The ideal nature of the required nutritional support needs further research, taking into consideration this unique group’s risk of NEC, metabolic imbalances, and other morbidities. 

Postnatal head growth as determined by the change in HC z-scores from birth to discharge, but mainly post-discharge and mid-term, has been correlated with long-term neurodevelopmental outcomes among preterm infants [22,33,34,35,36]. In the current study, the mean HC PNGF was mild in both groups, with a significant advantage for SGAs over AGAs. This difference most likely results from the higher rate of SGAs vs. AGAs (26.6% vs. 8.5%) experiencing an increase in their HC z-scores. While among the AGAs, HC PNGF correlated with weight changes, this correlation was not found among the SGAs. HC PNGF (moderate and severe) also did not correlate with early neonatal morbidities among the SGAs. In a large population-based study conducted among 12,992 Israeli preterm infants born at <1500 gr, the rate of severe and moderate HC PNGF at discharge was very similar to that found in the current study: 1.7% and 8.6%, respectively, among the SGA group compared with 5.1% and 23.7% among the non-SGA group. In that study, neonatal morbidities were associated with postnatal head growth [37]. We speculate that this difference may be explained by a small number of infants among the SGAs experiencing HC PNGF and a low rate of morbidities among the AGAs.

In our research, we showed that while the SGAs were more likely to experience early neonatal morbidities, early respiratory disease, measured as need for oxygen support, was more likely to occur among the AGAs. The association between weight-for-GA status and respiratory morbidities has been the subject of controversy: while some studies demonstrate a lower rates of respiratory distress syndrome among SGAs, possibly due to increased in utero stress and accelerated lung maturation, others do not [38,39,40]. The results of the current study may partially explain this controversy: we suggest that the SGA group may reflect the stress-maturation theory, while among the AGAs the high rate of early but mild respiratory disease reflects the results of an emergent delivery without initiation of labor and its effect on the newborn lungs. This may be supported by the high rate of surgical deliveries (85.5%) and fetal indications for delivery in half of the cases (48.2%). Nevertheless, the duration of oxygen treatment was short and did not differ significantly between the SGA and AGA groups, and together with the fact that this cohort did not include very preterm infants (only >30 weeks of gestation), no conclusions can be drawn regarding the risk for bronchopulmonary dysplasia. 

To our knowledge, this study is the first to compare growth trajectories among a cohort of AGA/SGA twins. While previous studies have compared growth and outcomes of discordant twins, the specific purpose of this study was to assess the effect of being born SGA, according to the Fenton curves, on outcomes. The major advantage in comparing twins lies in decreasing the effect of various confounders, such as maternal, obstetric, genetic, and environmental factors. Not controlling for chronicity or for being identical cannot completely eliminate these confounders among other paired infants. Moreover, differences in nutritional management between the AGA and the SGA twin during the first days of life may have affected their growth outcomes. However, not only did the SGA infants regain their birth weight earlier, keeping infants nil per os for 72 h to prevent NEC is no longer a common practice. The early nutritional care given to SGA preterm infants is a modifiable factor with potential room for intervention. Further studies are needed to define the optimal caloric needs of these infants. Newly published data suggest that the postnatal growth of preterm infants should be compared with standards for postnatal growth derived from a cohort of accurately dated, uncomplicated pregnancies with adequately grown fetuses (i.e., INTERGROWTH-21st) [41]. The INTERGROWTH-21st enrolled 201 preterm singleton infants, of whom 28 born were at 33 weeks or earlier. A study that compared between the Intergrowth and the Fenton standards indicated that almost one out of every five cases assessed as PNGF according to Fenton standards was within the normal interval according to Intergrowth standards [42]. In contrast, one out of every four cases assessed as SGA according to the Intergrowth standards was within the normal interval according to Fenton standards. In the present study, we used the Fenton standards to allow for comparison with previous published data. 

The major limitation of this study lies in its retrospective nature. Moreover, the cohort is small and includes a wide range of gestational ages that reflect different populations. We tried to eliminate this factor by restricting inclusion criteria to infants born between 30 weeks and 34 weeks and 6 days who are all taken care of by the same medical team in the same NICU. Another limitation may lie in the choice of 36 weeks PMA as a time point for assessing PNGF. One may argue that for late preterm infants this may be too early to assess growth. As the best time point for PNGF (HC or weight) assessment has not been yet determined, and as 36 weeks PMA is the point at which infants may be discharged home, it seems to be reasonable for future intervention planning. Moreover, as this study was conducted in a single medical center, generalization of the data is limited. Nonetheless, the rates of PNGF in the present study cohort were similar to those published by others, implying the relevance of its results as well as the need for further study of early growth characteristics and their associations with long-term outcomes among preterm SGA infants. 

## 5. Conclusions

To summarize, the present study demonstrates that although AGA and SGA preterm infants had a similar weight PNGF, the SGA infants were subject to an extreme catabolic state resulting in severe malnutrition when they reached 36 weeks PMA. Using ∆ z-score to assess PNGF among SGA preterm infants may lead to underestimating this severe condition. Further studies are needed to assess the best measurement associated with long-term outcomes among the SGA group. We also demonstrated that time to regain birth weight is associated with weight PNGF among SGA infants. This finding suggests that this group may benefit from unique early nutritional support. 

## Figures and Tables

**Table 1:** Maternal demographics and pregnancy characteristics.

Maternal Characteristics	*n* = 83
Median maternal age at delivery, years (min–max)	32.5 (21–52)
In vitro fertilization, *n* (%)	41 (49.4)
First pregnancy, *n* (%)	49 (59.0)
Gestational diabetes, *n* (%)	10 (12.0)
Prenatal steroids, *n* (%)	68 (81.9)
Maternal hypertension during pregnancy, *n* (%)	14 (16.9)
Fetal indication for delivery, *n* (%)	40 (48.2)
Spontaneous preterm labor, *n* (%)	32 (38.6)
Surgical delivery, *n* (%)	71 (85.5)
Median gestational age, weeks (min–max),	33.6 (30.9–34.9)
Discordance < 10%, *n* (%)	3 (3.6)
Discordance 11–15%, *n* (%)	9 (10.8)
Discordance 16–20%, *n* (%)	14 (16.9)
Discordance ≥ 20%, *n* (%)	57 (68.7)

Data presented as median (minimum–maximum), number (percentile).

**Table 2:** Infant characteristics and morbidities (*n* = 83).

	SGA	AGA	*p* Value
Male, *n* (%)	48 (57.8)	49 (59.0)	0.077
Minimal weight loss in % mean (SD)	7.6 (13.1)	8.8 (3.7)	0.404
Apgar 1 < 6, *n* (%)	10 (12.0)	4 (4.8)	<0.001
Hypoglycemia (<45 mg/dL within the first 72 h), *n* (%)	12 (14.5)	3 (3.6)	0.009
Admission temperature, mean (SD) (Celsius)	35.4 (0.7)	35.8 (1.3)	<0.001
Day of life to birth weight, mean (SD)	9.1 (4.5)	12.0 (4.6)	<0.001
Duration of antibiotics (d), mean (SD)	4.0 (5.7)	3.1 (5.2)	0.253
Hospitalization duration (d), mean (SD)	37.6 (19.7)	23.2 (11.7)	<0.001
Duration of fluids infusion (d), mean (SD)	9.8 (9.1)	5.1 (4.8)	<0.001
Day of first enteral feeding	3.1 (3.3)	2.0 (1.1)	0.007
Total parenteral nutrition, *n* (%)	65 (83.5)	48 (60.8)	<0.001
Total parenteral nutrition (d), mean (SD)	9.0 (8.5)	4.3 (4.8)	<0.001
Any breastmilk at day of life 14, *n* (%)	67 (71.3)	64 (68.1)	0.581
Any breastmilk at 36 weeks, *n* (%)	60 (63.2)	60 (63.2)	1
Proven sepsis, *n* (%)	16 (19.3)	3 (3.6)	0.002
Oxygen treatment, *n* (%)	22 (26.5)	37 (44.6)	0.006
Duration of oxygen treatment (d),	2.6 (11.9)	2.2 (3.4)	0.733
Oxygen at 36 weeks, *n* (%)	2 (2.4)	0	-
Necrotizing enterocolitis, *n* (%)	4 (4.8)	1 (1.2)	0.375
Severe morbidity *, *n* (%)	19 (22.9)	4 (4.8)	0.001

* Severe morbidity includes any of the following: oxygen requirement at 36 weeks GA, proven NEC, IVH > grade 2, PVL, surgical procedure, sepsis. SD: Standard deviation, d; days, NEC: necrotizing enterocolitis; IVH: intraventricular hemorrhage; PVL: periventricular leukomalacia.

**Table 3:** Infant growth parameters (*n* = 83).

Weight Characteristics	SGA	AGA	*p* Value
Birth weight (g), median (Min–Max) Mean (SD)	1284 (656–1830) 1291 (289)	1906 (1192–2625) 1909 (304)	<0.001
Birth weight (g), *n* (%)			
>1500	22 (26.5)	74 (89.2)	
1000–1499	47 (56.6)	9 (10.8)	<0.001
<1000	14 (16.9)	0	
Birth weight z-score, mean (SD)	−1.90 ± 0.47	−0.33 ± 0.50	<0.001
Weight at 36 wks (g), mean (SD)	1548 ± 257	2138 ± 234	<0.001
Weight at 36 wks z-score, mean (SD)	−2.80 ± 0.72	−1.29 ± 0.58	<0.001
Weight at 36 wks < 10%, *n* (%)	83 (100)	44 (53.0)	-
Weight at 36 wks < 3%, *n* (%)	72 (90.0)	10 (12.5)	<0.001
∆ z score weight from birth to 36 weeks, mean (SD)	−0.90 ± 0.46	−0.96 ± 0.44	0.247
∆ z score decrease of 0 to 0.99	55 (66.3)	50 (60.2)	
∆ z score decrease of 1 to 1.99	25 (30.1)	31 (37.3)	0.012
∆ z score decrease of ≥ 2	3 (3.6)	2 (2.4)	
HC at birth z-score, mean (SD)	−1.03 ± 2.42	0.26 ± 0.74	<0.001
HC at 36 wks z-score, mean (SD)	−1.67 ± 0.85	−0.51 ± 0.65	<0.001
HC at birth < 10%, *n* (%)	47 (57.3)	1 (1.2)	<0.001
HC at 36 wks < 10%, *n* (%)	52 (63.4)	9 (11.0)	<0.001
∆ z score HC from birth to 36 weeks, mean (SD)	−0.39 ± 0.72	−0.76 ± 0.66	0.001
∆ z score decrease of 0 to 0.99	47 (59.5)	55 (67.1)	
∆ z score decrease of 1 to 1.99	6 (7.6)	17 (20.7)	
∆ z score decrease of ≥2	5 (6.3)	3 (3.7)	0.395
Increased	21 (26.6)	7 (8.5)	

SD: standart deviation, HC: head circumference; AGA: appropriate for gestational age; SGA: small for gestational age; wks: weeks; g: gram.

**Table 4:** Unadjusted and adjusted multinomial logistic regression for estimating risk of moderate or severe weight PNGF among SGA and AGA infants.

	SGA				AGA			
	Risk of Moderate or Severe PNGF (Unadjusted)		Risk of Moderate or Severe PNGF (Adjusted)		Risk of Moderate or Severe PNGF (Unadjusted)		Risk of Moderate or Severe PNGF (Adjusted)	
	OR	95% CI	OR	95% CI	OR	95% CI	OR	95% CI
Gestational age	**0.30**	**0.18–0.52**	**0.26**	**0.0–0.69**	0.70	0.47–1.06	0.49	0.20–1.21
Birth weight	**0.99**	**0.99–0.99**	1.01	0.99–1.00	1.00	0.99–1.00	1.01	1.00–1.01
Gender	**2.53**	**1.00–6.42**	4.28	0.97–18.80	1.36	0.56–3.32	0.27	0.07–1.02
Sepsis	**3.25**	**1.06–9.97**	3.01	0.63–14.47	-	-	-	-
Oxygen treatment	**5.88**	**2.05–16.85**	1.78	0.38–8.42	2.41	0.98–5.93	1.63	0.38–1.02
Fluid infusion duration	**1.09**	**1.03–1.16**	1.05	0.97–1.15	**1.22**	**1.07–1.38**	1.23	0.97–1.57
Days to regain birth weight	**1.21**	**1.05–1.38**	**1.22**	**1.00–1.48**	**1.40**	**1.19–1.64**	**1.34**	**1.12–1.61**

OR: Odds ratio, CI: confidence interval, PNGF: postnatal growth failure. Bold numbers indicate statistically significant (less than 0.05).
